# Correction

**DOI:** 10.1080/19420862.2025.2463770

**Published:** 2025-02-06

**Article title**: With great power, comes great responsibility: The importance of broadly measuring Fc-mediated effector function early in the antibody development process

**Authors**: Crescioli, S., Jatiani, S., & Moise, L.

**Journal**: *mAbs*

**DOI**: https://doi.org/10.1080/19420862.2025.2453515

The author has noticed an error in the captions of Figures 2 and 3, which were incorrectly typed in the published article.

The author has requested that the current captions of Figures 2 and 3 be replaced with the updated versions provided below, as they more accurately reflect the original intentions.

**Figure 2:** Fc-mediated effector functions: The schematic depicts the variety of cells expressing Fc receptors (FcRs) and the different types of Fc-mediated effector functions. Depending on the affinity for each FcR, antibodies can engage different type of cells and mediate different effector functions. Direct effector functions include: complement-dependent cytotoxicity (CDC), antibody-dependent complement deposition (ADCD), antibody-dependent cell cytotoxicity (ADCC), antibody-dependent cell phagocytosis (ADCP) and, for pathogen targeting antibodies, antibody dependent mucin binding (ADMB). Antibodies can also mediate indirect effector functions such as antigen presentation with priming of both CD4+ and CD8+ T cells. ADCD can also facilitate phagocytosis by cells expressing complement receptor CR3. Engagement of FcRs on immune cells can also result in cytokine release, or antibody dependent enhancement (ADE) of infection or disease (not depicted in this schematic). Excessive cytokine release and ADE of infection or disease are Fc-mediated functions that pose safety issues. The boxes underneath each cell name show the expressed FcRs, * is for inducible expression, # is for expression in a subset of the cells ^8,9,23^. The red boxes show the types of Fc-mediated effector function. Unlabelled molecules and cells are described in the key box on the right.

**Figure 3:** Antibody design: schematic depicting the variables to consider during antibody design (boxes on the left) and the effect of Fab and Fc design on antibody function due to Fab/Fc interdependence (schematic on the right).

